# Hospital Meetings

**Published:** 1903-06-13

**Authors:** 


### HOSPITAL MEETINGS.

### THE HOSPITAL FOR SICK CHILDREN, GREAT ORMOND STREET.

His Grace the Duke of Fife took the chair at the annual meeting of governors of this hospital on May 25th, which was by no means well attended.

The Duke of Fife, in moving the adoption of the report, said that on the whole they might congratulate themselves on the general position of the hospital. For some time past those interested in it must have felt that there was one blot on the hospital, one point upon which they were open to criticism, and that was the condition of the out-patient department, but all their difficulties had been dispelled by Mr. Astor's magnificent gift of £50,000. This splendid donation would enable them to build an out-patient department in every way worthy of the work done by the hospital. It was difficult, his Grace went on, to find adequate words in which to express the profound gratitude they all felt to Mr. Astor for so nobly coming to their rescue. They must, however, bear one point in mind, and that was that this sum was practically ear-marked and could be devoted to no other purpose than that of building an out-patient department. Referring to the Imperial Coronation Bazaar which was held last summer in the Royal Botanic Gardens, the Duke of Fife considered this to have been an immense success, thanks to the strenuous exertions of the ladies and gentlemen who took so much interest in it. The net proceeds amounted to nearly £19,000, and this sum had enabled them to pay off a portion of the debt on the hospital and also to meet last year's and this year's terrible deficit of £7,C00. It was to meet this deficit, which was always staring them in the face, that they were compelled to have recourse to all sorts of methods of raising money, and if it were not for the legacies those who were responsible for the finances might almost despair as to the future of the hospital. There was one satisfactory feature in the report, said his Grace, and that was the growth of the endowment fund. In ten years it had increased by nearly £40,000, and this, compared to the increase in the ten years previously, was enormous: if the fund only continued to grow at its present rate during the next ten years it should reach £200,0C0, and this would place them beyond the necessity of making efforts to raise money and never-ending appeals to the public. The Duke considered that the time of the finance committee was far too much taken up with begging, and apart from that side of the question there was also the great anxiety which everyone interested in the hospital was under every year, as to whether they would be able to carry on their good work on the same scale. The paramount necessity for increasing the endowment fund should be impressed upon everyone, for by its means alone could the terrible weight of anxiety that rested on everyone connected with the hospital be lightened.

The adoption of the report was seconded by Mr. Arthur Lucas, chairman of the committee of management, who also proposed the confirmation of the proceedings of the committee of management. Mr. Lucas prefaced his remarks by an expression of gratitude, on behalf of the committee of management, to Mr. Astor for so nobly solving their most troublesome problem. But this gift would involve them in additional responsibility and expense. Mr. Lucas here mentioned a remark that had been made to him when Mr. Astor's donation was first made known, to the effect that for the future it was to be supposed that nothing further would be heard of appeals for help from that hospital; but the speaker went on to show how mistaken such a notion was. One additional expense entailed by the gift would be the higher rates they would have to pay for larger buildings. The question of rates, said Mr. Lucas, was one that affected all the hospitals. In the first 10 years of this hospital's existence they paid in rates the sum of £55; this figure went on rising as follows:—£98, £313, £426, £636, until last year it reached £903; that is, for every pound given by the subscribers 1s. went to the rates. It was the duty of the State to grapple with this question. It was objected that if the rates of the hospitals were diminished they would be increased for other people, to which their answer was—a good thing too! Sir Henry Burdett some years ago mentioned in a speech that he had taken the trouble to make an investigation of the inhabitants of Belgrave Square, and out of the whole number of dwellers in that square there were only two who were subscribers or donors to any of the London hospitals. The item of rates, Mr. Lucas went on, was one that was beyond their control, and there were others in the same category; for instance, cod-liver oil, of which they consumed five tons a year, had risen in price from 5s. 6d. to 20s. per gallon by reason of the failure of the cod-fisheries. It was sometimes said that this hospital was extravagant in its expenditure, but this assertion Mr. Lucas denied *in toto*, and said that it was the one aim of the committee to maintain the hospital in as economical and efficient a manner as possible.

The resolution was seconded by Mr. Edmund Owen, who took the opportunity of stating the entire satisfaction the staff felt with the management.

Lord Aberdare moved a resolution for the re-election of officers for the coming year, and remarked what a great advantage it was to the hospital to have as their president the Duke of Fife, not only on account of his connection with the Royal Family, but also by reason of his assiduous attention to his duties. Other business having been transacted, Mr. John Murray moved a vote of thanks to Mr. Astor for his munificent gift, and said there was one aspect of the subject that came home to all their hearts, and that was that Mr. Astor had been led to make this gift because his little daughter, in whose memory the donation was made, had been attended by nurses from their hospital.

This resolution was seconded by Mr. C. D. Kemp-Welch.

Mr. Arthur Lucas, in moving a vote of thanks to the Duke of Fife for taking the chair, said that his Grace in speaking of the success of the bazaar, had omitted to mention the action he had taken in asking the Queen to open it, thereby securing for it so great brilliancy and success.

The Duke of Fife in responding said that it was always a labour of love to him to do all he possibly could for the hospital, and with regard to the bazaar, he assured the meeting that the Queen took the greatest possible interest in the hospital, and it was not a very difficult task to persuade her to open the bazaar. There was one terrible moment, his Grace went on, at the time of the King's illness, when he had feared the Queen would be unable to attend, but happily this disappointment was spared them.

The hospital was lucky, as appeared in the report, in securing the large sum of £1,500 from the King's Fund besides £1,054 from the Sunday Fund, and £270 from the Saturday Fund.

### HOSPITAL FOR CONSUMPTION, BROMPTON.

On May 28th the annual meeting of Governors was held at the hospital, the Earl of Derby, K.G., presiding. His lordship, in moving the adoption of the report, alluded in sympathetic terms to the losses the hospital had sustained through the deaths of Mr. W. S. Deacon, the treasurer, and the Rev. H. E. Warner, and the retirement of Dr. Henry Green after 27 years' work at the hospital. Continuing, Lord Derby said that it was one of the chances of life that an old institution like this which in early days had done so much to combat disease and to promote the science of health should in course of time be equalled and temporarily surpassed by other hospitals, which perhaps advertised more freely and therefore obtained a better hold on public attention. His lordship made an earnest appeal to all connected with the hospital to do their best to let people know that this institution was by no means lagging behind, but was endeavouring to bring itself up to the level of the most perfectly equipped modern hospitals, and was doing as good work now as in the commencement of its career. Of late years it had been found necessary for the best treatment of this disease to go into fresher scenes where more light and air were available. They had now an increasing number of formidable rivals; it was a noble rivalry, but it did undoubtedly tend to take away from them some subscribers and a certain amount of interest. Almost every county was now establishing sanatoria, and appeals were being issued for their support. They were not now exclusive recipients of public bounty, but they must not be discouraged, but must nerve themselves to greater efforts and to still further sacrifices as difficulties arose. They must all have been struck, Lord Derby went on, by the formidable discrepancy between the receipts and expenditure, and it would seem even more formidable were it not that they hoped that the expenditure of capital upon the construction of the branch home at Heatherside would in time to come not only bring its own reward in point of health, but would form a valuable adjunct to the hospital. It was only of recent years that the advantages of light and fresh air in the treatment of consumption had been grasped by the public imagination. In two directions much remained to be done, firstly in the segregation of those in the early stages of this disease, and secondly in the removal from the hospitals of those for whom there were no chances of recovery. On the whole, the work of the hospital went on steadily and well, but they asked for greater help in order to enable them to do much more next year. In future they would be compelled to make even larger demands on the purses of their subscribers. They must all try and interest more friends in the work of the hospital, and to let it be known that at Heatherside they were doing their duty by constructing what was a necessary adjunct to the hospital. It was only by letting their work be known that they could hope to hold their own against so many other societies.

The resolution was seconded by Mr. T. P. Beckwith, chairman of the committee of management, who, referring to the branch at Heatherside, said that they hoped in a few months to be able to show an institution in every way adequate to carry out the treatment that was acknowledged to be best for consumptive patients.

The Rev. F. A. Minnitt next made some comments on the length of time that elapsed between the presentation of a letter to the hospital and the admission of a patient, and said that on more than one occasion lives had been lost by this delay of two or three months.

The usual business having been transacted, a vote of thanks to the Chairman was proposed by Mr. R. B. Chapman, who, replying to Mr. Minnitt, said that if the hospital were large enough to permit the admission of any patients at any moment such delays could not occur. They recognised and deplored the fact that lives were often lost because the patients could not gain admission immediately.

The chief point of interest in the report recorded the satisfactory progress in the building of the country branch for open-air treatment and convalescent home at Heatherside, near Frimley. The committee regretted that subscriptions for this work were not coming in as they had expected, and earnestly appealed for more help.

The Earl of Derby has contributed £1,000 towards this object.

### MIDDLESEX HOSPITAL.

The Quarterly Court of Governors of this hospital was held on Thursday, May 28th, Major-General Lord Cheylesmore being in the chair. A précis of the transactions of the Weekly Board was then read to the meeting, the chief points of interest in which were as follows:—

The opening of the new buildings forming the extension of the nurses' home, which, affording additional accommodation to the extent of 37 bedrooms, had permitted a much-needed increase of 25 nurses. It had at the same time been thought advisable to appoint an assistant matron, home sister, and assistant to the home sister, and to grant extra allowances of leisure time to the ward sisters and staff nurses.

In consequence of the increase of work in the new clinical laboratory it had been decided to appoint a director of cancer research, and also to establish a cancer research scholarship of £105 per annum.

The Quarterly Court were asked to authorise the sale of stock to the amount of £10,000.

The adoption of the report was moved by Sir Arthur Watson, K.C., who, referring to the new buildings for the nurses, said it had been suggested that in this hospital the nurses were too hard worked, but after inquiry they were not of opinion that such was the case; recognising however how trying and responsible was the work entrusted to them, they had taken the opportunity of providing additional accommodation for 25 sisters and nurses, and trusted before long to be able to make all the necessary appointments With regard to the new officer of which it was desired establish, Sir Arthur said they hoped to secure the services of a very good man for this post of director of cancer research. The new electrical and light department had been worked with great success. This hospital, the speaker went on, was particularly interested in investigations as to the origin of cancer, one of its founders having left a considerable sum for this purpose. Although much had been done in this country in the way of investigation of this disease, there was no place where it was carried out with so much success as at the Middlesex Hospital, where there was always a large number of cancer patients. Sir Arthur Watson ended by paying a tribute to the efficient work performed by the staff.

The resolution was seconded by Mr. Andrew Clarke who, on behalf of the staff, thanked the board for having dealt with the nursing question which had been outstanding for so long. With regard to the electrical and light department, Mr. Clarke said it had not yet been working very long, but had already shown that it would be of great use in the treatment of skin diseases and also of cancer. A great many cancer patients had been very much relieved and the disease checked by the light treatment.

The usual business having been transacted, the proceedings closed after a vote of thanks had been passed to Lord Cheylesmore for presiding.

### HOSPITAL FOR EPILEPSY AND PARALYSIS.

The annual meeting of the Hospital for Epilepsy and Paralysis was held on Tuesday, May 26th, in the board-room of the new building in Maida Vale. Mr. Charles Drummond, treasurer, presided. Owing probably to the near approach of the opening ceremony, fixed to take place on June 13th, the meeting was more formal and less fully attended than is usual at this institution, some 13 being present, of whom several were ladies. The report, read by the secretary, Mr. H. Howgrave Graham, showed that while the annual subscriptions in 1902 amounted to £295, the outgoings in ground rent and local rates alone exceeded that sum by £136, thus proving the urgent need for additional support, to enable the committee to cope with their largely increased responsibilities. It is hoped that H.R.H. Princess Louise, his Grace the Duke of Argyll, and the Lord Bishop of London will take part in the opening ceremony.

### THE NORTH-EASTERN HOSPITAL FOR CHILDREN.

The thirty-fifth annual meeting of Governors of this hospital took place on Thursday, the 28th ult., in the new board-room. Mr. Walter Johnson, Mayor of Hackney, presided, and in moving the adoption of the report and accounts for 1902 recalled the small beginnings of the hospital as a dispensary for women and children in Virginia Row. Established by Miss Mary Phillips and her late sister, Mrs. Alexander Fox, in 1867, the institution had grown in size and importance, and to-day showed an imposing structure immediately facing Hackney Road, which would shortly be completed and opened with 54 beds. It was hoped, Mr. Johnson added, that the opening ceremony would be performed by a member of the Royal Family. With regard to the financial position, the committee had promises of £1,900, which, with £1,850 in hand, made a total of £3,750. There still remained £9,000 to find before the new building would be free of debt. The total cost, including the site, was £32,000, most of which had been paid off. The remaining portions of the scheme, comprising a nurses' home and laundry, a new out-patient department, and a separate isolation block, which would entail the expenditure of another £25,000, would be proceeded with as soon as possible, all these buildings being essential to the efficiency of the daily work and to the increased economy in administrative expenditure which the committee were anxious to secure. It must be understood that the annual cost for maintenance would be increased by about £3,000, and the best efforts of the Governors must be directed towards increasing the reliable income in subscriptions and donations.

The Rev. W. G. Morcom, in seconding the motion, spoke of the devoted work of the Hackney teachers, who so largely contributed to the success of a bazaar at the Baths, as a result of which nearly £1,700 was handed over to the hospital.

Several speakers alluded to the retirement, after 25 years' work, of the matron, Miss Corno; her place had been filled, with every prospect of satisfaction, by Miss A. M. Bushby.

